# Heterodimerization of Glycosylated Insulin-Like Growth Factor-1 Receptors and Insulin Receptors in Cancer Cells Sensitive to Anti-IGF1R Antibody

**DOI:** 10.1371/journal.pone.0033322

**Published:** 2012-03-16

**Authors:** Jun Gyu Kim, Min Jueng Kang, Young-Kwang Yoon, Hwang-Phill Kim, Jinah Park, Sang-Hyun Song, Sae-Won Han, Jong-Wan Park, Gyeong Hoon Kang, Keon Wook Kang, Do Youn Oh, Seock-Ah Im, Yung-Jue Bang, Eugene C. Yi, Tae-You Kim

**Affiliations:** 1 Cancer Research Institute, Seoul National University, Seoul, South Korea; 2 Department of Internal Medicine, Seoul National University, Seoul, South Korea; 3 WCU Department of Molecular Medicine and Biopharmaceutical Science, Graduated School of Convergence Science and Technology, Seoul National University, Seoul, South Korea; 4 Department of Pharmacology, Seoul National University, Seoul, South Korea; 5 Department of Pathology, Seoul National University, Seoul, South Korea; 6 Department of Nuclear Medicine, College of Medicine, Seoul National University, Seoul, South Korea; University of Munich, Germany

## Abstract

**Background:**

Identification of predictive biomarkers is essential for the successful development of targeted therapy. Insulin-like growth factor 1 receptor (IGF1R) has been examined as a potential therapeutic target for various cancers. However, recent clinical trials showed that anti-IGF1R antibody and chemotherapy are not effective for treating lung cancer.

**Methodology/Principal Findings:**

In order to define biomarkers for predicting successful IGF1R targeted therapy, we evaluated the anti-proliferation effect of figitumumab (CP-751,871), a humanized anti-IGF1R antibody, against nine gastric and eight hepatocellular cancer cell lines. Out of 17 cancer cell lines, figitumumab effectively inhibited the growth of three cell lines (SNU719, HepG2, and SNU368), decreased p-AKT and p-STAT3 levels, and induced G 1 arrest in a dose-dependent manner. Interestingly, these cells showed co-overexpression and altered mobility of the IGF1R and insulin receptor (IR). Immunoprecipitaion (IP) assays and ELISA confirmed the presence of IGF1R/IR heterodimeric receptors in figitumumab-sensitive cells. Treatment with figitumumab led to the dissociation of IGF1-dependent heterodimeric receptors and inhibited tumor growth with decreased levels of heterodimeric receptors in a mouse xenograft model. We next found that both IGF1R and IR were N-linked glyosylated in figitumumab-sensitive cells. In particular, mass spectrometry showed that IGF1R had N-linked glycans at N913 in three figitumumab-sensitive cell lines. We observed that an absence of N-linked glycosylation at N913 led to a lack of membranous localization of IGF1R and figitumumab insensitivity.

**Conclusion and Significance:**

The data suggest that the level of N-linked glycosylated IGF1R/IR heterodimeric receptor is highly associated with sensitivity to anti-IGF1R antibody in cancer cells.

## Introduction

With its secreted ligands, IGF1 and IGF2, Insulin-like growth factor 1 receptor (IGF1R) is highly expressed in many human cancer cells, including gastric (GC) and hepatocellular carcinoma (HCC) [1]–[5]. As a result, a variety of strategies inhibiting the IGF1R signaling pathway have been developed over the past two decades [6]. Among these, an anticancer therapeutic strategy using fully humanized antibodies has become an important research focus [7], because it has great potential for becoming successful anti-cancer therapeutics that could effectively inhibit cancer cell proliferation with low toxicity and provide clinical benefits when administered in combination with chemotherapy [8]–[14]. A fully humanized anti-IGF1R monoclonal antibody (figitumumab) has been tested in phase III clinical trials; however, no statistically significant improvement was demonstrated by administering figitumumab along with standard chemotherapy to patients with advanced non-small cell lung cancer (NSCLC) [15].

Many studies have shown that the A isoform of insulin receptor (IR) is abnormally overexpressed in various cancer types and might promote tumor growth [16]–[19]. This IR shares a high sequence homology with IGF1R, particularly within the intracellular kinase domain [7], [20]. IR pro-receptors can form heterodimeric receptors (HRs) with IGF1R pro-receptors post-translationally, prior to cleavage to generate two extracellular alpha subunits and two beta subunits that contain extracellular, transmembrane, and tyrosine kinase domains [21]. Therefore, when cells co-express IGF1R and IR, the pro-receptors can heterodimerize to create IGF1R/IR HRs [22]–[24]. These HRs may also be overexpressed in various tumor cells and specimens as a result of both IGF1R and IR overexpression [2], [25], [26]. Consequently, the relative abundance of IRs affects IGF system activation through HRs, which responds to both insulin and IGFs [27]–[29]. In cancer cells with high levels of IGF1R/IR HRs, IGF1 and IGF2 activate various downstream signaling pathways through heterodimeric receptors rather than through homodimeric IGF1Rs [30].

A number of studies have tried to identify predictive biomarkers with preclinical and clinical relevance [15], [31], [32]. Identification of predictive biomarkers for monitoring the efficacy of IGF1R targeted therapy for appropriate patients, however, is still needed. In the present study, we demonstrated that figitumumab possesses a high affinity for IGF1R/IR heterodimeric receptors as well as IGF1 homodimer receptors and inhibits the IGF/IGF1R signaling axis in gastric cancer and hepatocellular carcinoma cells. In addition, our data showed that functional membrane-bound IGF1R/IR heterodimeric receptors play a major role in IGF1 signaling [26], [33] and therefore may serve as biomarkers for predicting sensitivity to anti-IGF1R antibody.

## Results

### Anti-proliferative effect of figitumumab

**Figure 1:**
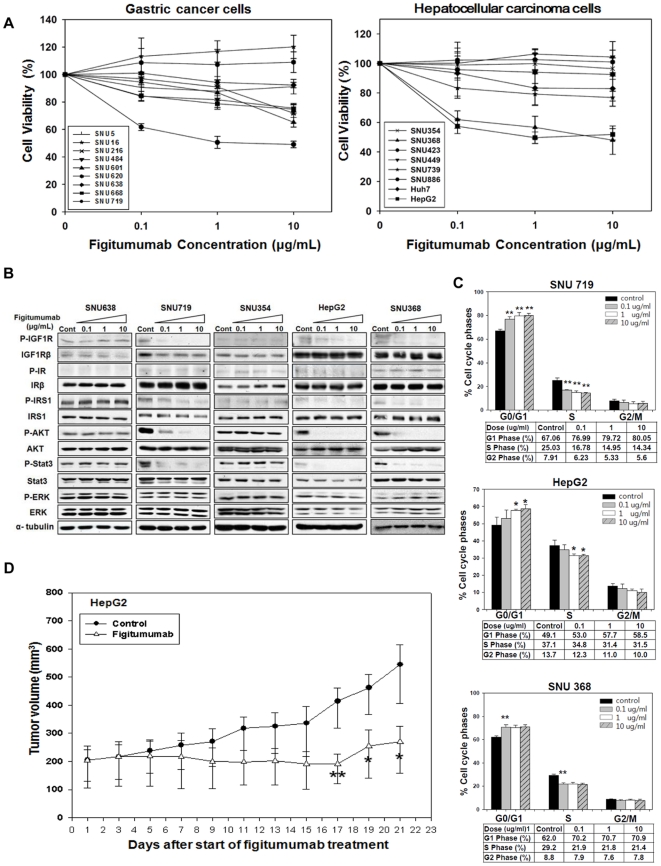
Anti-proliferative effect of figitumumab. A) Analysis of the anti-proliferative effect of figitumumab on gastric and hepatocellular carcinoma cells. Two groups of cancer cells, including nine gastric cancer cell lines and eight hepatocellular carcinoma cell lines, were treated with increasing concentrations of figitumumab (0, 0.1, 1, and 10 µg/mL) for 120 h to inhibit the growth of the control cells by 30%. Cell proliferation was assessed by an MTT assay. Six replicate wells were used for each analysis, and at least three independent experiments were conducted. Data from replicate wells are presented as the mean of the remaining cells. Bars = ±SE. B) Effect of figitumumab on the IGF1R signaling pathway. Immunoblotting analysis was performed to observe the dose-response effect of figitumumab (0.1–10 µg/mL) on IGF1R signaling. SNU638, SNU719, SNU354, HepG2, and SNU368 cells were exposed to increasing concentrations of figitumumab for 72 h. The levels of proteins associated with the IGF1R pathway and their activated forms were analyzed. Differences relative to the control are shown. In each panel, representative blots from three independent experiments are shown. C) Effect of figitumumab on the cell cycle distribution. Figitumumab-sensitive cells (SNU719, HepG2, and SNU368) were treated with increasing concentrations of the drug [0 µg/mL (black solid bar), 0.1 µg/mL (gray solid bar), 1 µg/mL (white bar), and 10 µg/mL (dark gray hatched bar)] for 48 h and then stained with propidium iodide, and analyzed by flow cytometry. The percentage of cells in the G_0_/G_1_, S, and G_2_/M phases are shown. Columns represent the mean of three independent experiments; Bars = ±SE. *P-values <0.05, **P-values <0.01. D) Effect of figitumumab on tumor growth in mice bearing HepG2 xenografts. HepG2 cells (1×10^7^) were injected into the right flank of nude mice (n = 5). Treatment with figitumumab (125 µg/mL [6.3 mg/kg body weight], once per week for 3 wk) was initiated once the tumor volume had reached 200 mm^3^. No significant body weight loss was observed during the course of the study. The tumors were measured with calipers at regular intervals. Solid circles = treatment with vehicle control alone (control), Open triangles = treatment with figitumumab. Differences between the two groups (tumor sizes of the control mice and those of mice treated with figitumumab) were compared from day 17 until the end of the treatment period (day 21) using a two-sided Student’s *t* test. *P-values <0.05; **P-values <0.01 versus control.

As a first step, we assessed the anti-proliferative effect of figitumumab, a monoclonal antibody that prevents ligands from binding to IGF1R [12], on 17 cancer cell lines (Figure 1A). Some cells considered to be sensitive to figitumumab, such as SNU719, HepG2, and SNU368, showed a dose-dependent decrease of cell viability; IC_30_ value of figitumumab (growth inhibitions of ∼30%) for each cell line were 0.063 µg/ml, 0.062 µg/ml, and 0.047 µg/ml, respectively (Table 1).

**Table 1:** Anti-proliferative effect of figitumumab in gastric cancer and hepatocellular carcinoma cells.

GC cells	Figitumumab IC^30^ (µg/mL)	HCC cells	Figitumumab IC^30^ (µg/mL)
SNU5	>10	Huh7	>10
SNU16	>10	HepG2	0.047 ± 0.096
SNU216	>10	SNU354	>10
SNU484	>10	SNU368	0.062 ± 0.02
SNU601	4.313 ± 0.327	SNU423	>10
SNU620	>10	SNU449	>10
SNU638	>10	SNU739	>10
SNU668	>10	SNU886	>10
SNU719	0.063 ± 0.098		

NOTE: The IC_30_ values of figitumumab were determined by MTT assays,

The IC_30_ value is the drug concentration required for 30% cell proliferation inhibition.

GC = gastric cancer; HCC = hepatocellular carcinoma.

### Figitumumab disrupts IGF1R signaling mainly through AKT and STAT3 pathways and induces G_1_ arrest

To examine the mechanism through which figitumumab inhibits cell proliferation, we examined whether there were any differences in downstream signaling between sensitive and resistant cells in the presence of serum after long-term treatment with serial doses of figitumumab (Figure 1B). In this experiment, only figitumumab-sensitive cells exhibited markedly decreased levels of p-AKT and p-STAT3 in a dose-dependent manner; however, there were no changes in the levels of p-ERK. We also observed that figitumumab decreased the level of total cellular IGF1R in SNU719 cells, suggesting that down-regulation of this receptor might represent an antibody-mediated degradation process [8]. We investigated whether figitumumab induced down-regulation of IR expression as well as IGF1R levels in other cells at several different time points. Interestingly, figitumumab caused the rapid decrease of total IGF1R levels after 3 hour in SNU668 and SNU739 cells (IR-negative cells) but did not significantly down-regulate either IR or IGF1R expression in the other cell lines (Figure S1). In contrast, figitumumab did not down-regulate pAKT, pERK, or pSTAT3 in resistant cells.

To determine IGF1R signal dependency in sensitive cells, we next performed experiments with siRNA to silence IGF1R expression (Figure S2A). The results indicated that IGF1R sequence-specific siRNAs induced profound IGF1R down-regulation without influencing IR expression and showed a clear correlation between the ability of siRNA and figitumumab to inhibit the phosphorylation of specific down-stream signals, such as p-AKT and p-STAT3, only in sensitive cells. We also investigated the effects of IGF1R knockdown on the proliferation of sensitive cells and confirmed that silencing IGF1R expression resulted in an anti-proliferative effect on sensitive cells (Figure S2B). In short, these results showed that the anti-proliferative effects of figitumumab are specifically mediated through the down-regulation of AKT and STAT3 signaling pathways rather than through the ERK signaling pathway in sensitive cells which have a strong IGF1R signaling dependency.

To further analyze the mechanisms through which figitumumab inhibited the proliferation and survival of cancer cells, we conducted a flow cytometric analysis (Figure 1C). Figitumumab induced a similar dose-dependent increase in the percentage SNU719, HepG2, and SNU368 cell in the G1 Phase. However, there was no increased rate of apoptosis (percent of sub-G1 cells; data not shown). This analysis showed that figitumumab decreased cell viability through cell cycle inhibition without inducing apoptosis.

### Antitumor activity of figitumumab in a xenograft tumor model

We next sought further evidence of figitumumab activity *in vivo* by using HepG2 to establish xenografts due to their sensitivity to figitumumab *in vitro*. To assess the effect of figitumumab on tumor growth *in vivo*, xenograft tumors were grown in athymic nude mice. As shown as shown in Figure 1D, repeated weekly administration of single dose of figitumumab (6.3 mg/kg body weight) to animals bearing HepG2 tumors resulted in substantial tumor growth inhibition for 21 d of figitumumab dosing and significantly inhibited tumor growth at day 17 (P<0.01). In addition, we tested the effect of figitumumab on IGF1R-related molecules after 1 d of figitumumab treatment. Figitumumab effectively reduced the levels of phosphorylated IGF1R and IRS1 (Figure S3A). Taken together, these data showed that treatment with a single dose of figitumumab effectively inhibited the growth of tumors by inhibiting IGF1R and IRS1 activation.

### Overexpressed IGF1R and IR form IGF1R/IR heterodimeric receptors in figitumumab-sensitive cells

**Figure 2:**
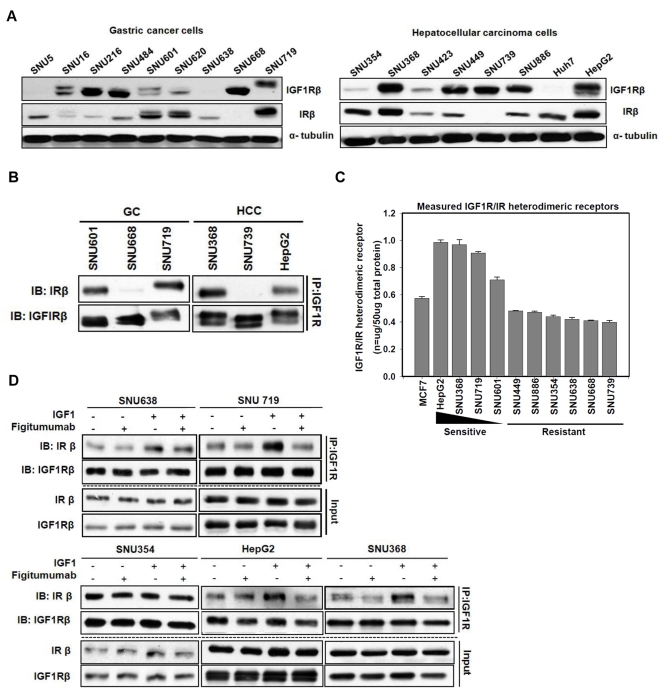
Analysis of IGF1R, IR, and IGF1R/IR HR levels in cancer cells sensitive to anti-IGF1R antibody. A) Immunoblot analysis of total IGF1Rβ and IRβ protein levels. Two types of gastric and hepatocellular carcinoma cells were harvested 24 h after plating and immunoblotting with anti-IGF1Rβ antibody, anti-IRβ antibody, and anti-α tubulin antibody was performed. For both types of cells, representative blots from three independent experiments are shown. B) Analysis of the presence of IGF1R/IR heterodimeric receptor (HRs) using immunoprecipitation. Total cellular proteins (1 mg) from cells were used for immunoprecipitation with anti-IGF1R antibody, separated by SDS-PAGE at constant voltage (80 V), and Western blotted with an anti-IRβ antibody. The blot was then stripped and reprobed with anti-IGF1Rβ antibody C) Quantitative analysis of IGF1R homodimer, IR homodimer, and IGF1R/IR heterodimer levels using an ELISA. Lysis buffer (100 µL) containing equal amount of proteins (50 µg/well) from 11 cancer cell lines including MCF7 cells (positive control) were plated on anti-IGF1R antibody-coated wells and detected with an anti-IR detection antibody. Anti-IR antibody-coated wells, IR protein standards, and the anti-IR detection antibody were used as standards in the heterodimeric receptor ELISA. Absorbance was measured at 450 nm. Values are expressed as the mean±SEM nanograms of receptor protein per 50 µg total protein. Cell lines are listed according to their sensitivity to figitumumab. Bars = ±SE. D) Effect of figitumumab on IGF1- mediated IGF1R/IR HRs. Cells were serum-starved for 24 h and then treated with figitumumab, IGF1, or left untreated. SNU719 cells were incubated with figitumumab (10 µg/mL) for 1 h at 37°C followed by stimulation with IGF1 (100 ng/mL) for 30 min. Immunoprecipitation was performed with an anti-IGF1R antibody and Western blotted. Input = total cell lysate without IP.

To identify a target for predicting sensitivity to figitumumab, expression of IGF1R related-proteins and downstream signaling molecules were analyzed in parallel by Western blotting. Interestingly, we found that figitumumab-sensitive cancer cells all overexpressed IGF1R; basal expression levels of IR were also much higher compared to that in other resistant cells (Figure 2A). Based on a recent report [31], we expected that figitumumab would specifically inhibit the growth of cells overexpressing IGF1R or its phosphorylated form, but not ones overexpressing IR because figitumumab does not bind to IRs [12]. However, IR protein levels were more responsive to figitumumab than any other protein. As shown in Figure 1A, the anti-proliferative effect of figitumumab was weaker in cells overexpressing only IGF1R, such as SNU668 and SNU739, than in cells overexpressing both IGF1R and IR. This finding suggested that different *in vitro* sensitivities of cells to figitumumab is associated with both IGF1R and IR levels which in turn can affect the level of IGF1R/IR heterodimeric receptors [2].

Since it is commonly known that IGF1R and IR can form heterodimers when both are co-overexpressed due to their highly homologous structures [2], we performed immunoprecipitation experiments to determine whether IGF1R interacts with IR to form heterodimer in figitumumab-sensitive cells. As shown in Figure 2B, cells overexpressing both IGF1R and IR, such as SNU719, HepG2, and SNU368, contained IGF1R/IR heterodimers. The SNU601 cell line, which showed modest sensitivity to figitumumab, also contained IGF1R/IR heterodimers. To determine whether figitumumab preferentially recognizes IGF1R/IR heterodimeric receptors in sensitive cells [12], we performed immunoprecipitation experiments using figitumumab as the antibody used for immunoprecipitation. Significant levels of both IGF1R and IR in figitumumab-sensitive cells were detected in figitumumab immunoprecipitates, suggesting that this antibody has a superior ability to recognize both the IGF1R homodimer and IGF1R/IR heterodimer predominantly in sensitive cells (Figure S4).

We also quantitatively measured IGF1R, IR, and IGF1R/IR HR levels using specific ELISAs with antibodies that specifically recognize IGF1R or IR and do not cross-react with each other. We compared 11 cancer cell lines, including MCF7 (Figure S5) which has been evaluated in a previous study [2]. The levels of IR ranged from 0.08 to 2.3 ng/50 µg total cellular proteins, and IGF1R levels ranged from 0.50 to 10.6 ng/50 µg total cellular proteins (Table 2). These results indicated that the expression of IGF1R and IR were similar, and most ELISA results correlated closely with the Western blotting results (Figure 2A). The cellular level of IGF1R/IR HRs ranged from 0.39 to 0.99 ng/50 µg total cellular proteins. The level of HRs was higher in sensitive cells than resistant cells (Figure 2C), suggesting that the expression level of the IGF1R/IR heterodimeric receptor significantly correlated with drug sensitivity.

**Table 2:** Summary of measured IGF1R homodimer, IR homodimer, and IGF1R/IR heterodimeric receptor values.

Cancer cell lines	Content of receptor (ng/50 µg protein)		
	IGF-1R	IR	HRs
GC			
SNU638	0.574 ± .01	0.725 ± .01	0.425 ± .01
SNU601	1.87 ± .03	1.923 ± .08	0.76 ± .01
SNU668	6.226 ± .05	0.178 ± .01	0.41 ± .003
SNU719	1.464 ± .04	2.293 ± .002	0.902 ± .01
HCC			
SNU354	0.499 ± .002	1.174 ± .039	0.446 ± .007
SNU368	3.188 ± .086	0.869 ± .008	0.953 ± .012
SNU449	6.143 ± .046	0.289 ± .012	0.482 ± .003
SNU739	10.567 ± .226	0.079 ± .005	0.397 ± .01
SNU886	2.766 ± .017	0.326 ± .024	0.47 ± .006
HepG2	5.633 ± .012	2.06 ± .102	0.985 ± .013
BC			
MCF7	14.903 ± .1	0.261 ± .01	0.535 ± .01

NOTE: Values are mean ± SEM nanograms of receptor protein/50 µg total protein.

Abbreviation: IGF1R = Insulin like growth factor 1 receptor; IR = Insulin receptor; HR = Heterodimeric receptor; GC = Gastric cancer; HCC = Hepatocellular carcinoma; BC = Breast cancer.

### Formation of IGF1 ligand-dependent IGF1R/IR heterodimer is inhibited by anti-IGF1R antibody

To further define the mechanism of anti-proliferative figitumumab activity related to IGF1R/IR heterodimer expression, we also examined changes in ligand-dependent heterodimeric receptor expression (Figure 2D). We found that the heterodimers bound to IGF1 ligands, but this IGF1 ligand-dependent formation was suppressed by figitumumab in sensitive cells. In SNU368, it appeared that figitumumab suppressed not only IGF1 ligands binding to the HRs, but also the expression of IGF1R/IR HRs in the absence of IGF1 ligand. Heterodimeric receptor levels in SNU638 and SNU354 cells, however, were relatively stable in the presence of figitumumab. Additionally, there was no detectable insulin-dependent heterodimer formation or dissociation due to figitumumab. Phosphorylation in response to 100 nM insulin was also not reduced by figitumumab (Figure S6). Taken together, the results from this experiment demonstrated that IGF1R/IR heterodimers responded well to IGF1, and blocking of IGF1 by figitumumab induced the down-regulation of IGF1 ligand-dependent IGF1R/IR heterodimer formation in the drug-sensitive cell lines.

### Selective overexpression of IR induces heterodimeric receptor formation and enhances the anti-proliferative effect of figitumumab

**Figure 3:**
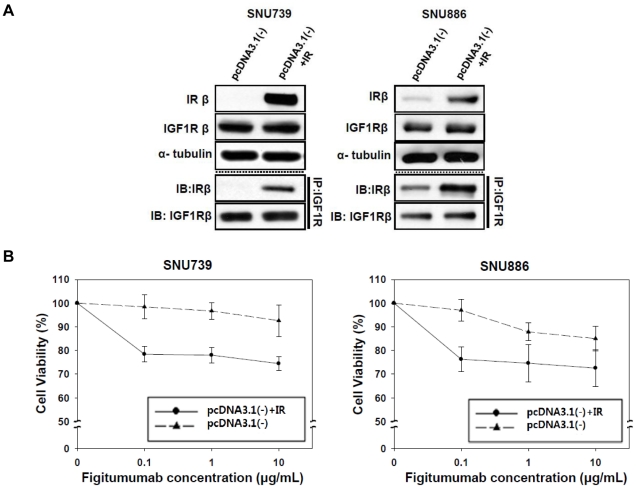
Effect of selective IR overexpression on HR levels and the anti-proliferative effect of figitumumab in IR-transfected cells. A) Effect of IR transfection on IGF1R/IR HR levels in IR-negative cell lines. Cells were transfected with the pcDNA3.1(-) expression vector containing wild-type IR cDNA. An equal amount of lysates from cells transfected with either the empty vector or pcDNA3.1(-) containing IR cDNA was subjected to immunoprecipitation with an anti-IGF1R antibody followed by Western blot analyses of IRβ and IGF1Rβ. B) Effect of IR-transfection on figitumumab sensitivity. Transfected cells were plated onto 96-well plates, treated with figitumumab for 5 d, and subjected to MTT assays. Solid triangle symbol with dashed lines = empty vector (pcDNA3.1-), Solid circle symbols with lines = pcDNA3.1(-) IR. Bar = ±SE. Mean values were derived from six replicates. Experiments were repeated in triplicate.

To evaluate whether the effect of figitumumab was restricted to SNU719, HepG2, or SNU368 cells, we performed studies in cells with low expression levels of IR, including SNU739 and SNU886 cells, transfected with pcDNA3.1-IR which induced high expression of IR. As shown in Figure 3A, IR expression levels in the transfected cells increased remarkably compared to cells transfected with the pcDNA3.1(-) empty vector. Moreover, IGF1R/IR HRs were also formed in the IR-transfected cells. To examine whether IGF1R/IR HR formation due to increased IR protein levels could enhance drug sensitivity, we performed MTT assays. The result showed that IR-transfected cells were more sensitive to the increased anti-proliferative effect of figitumumab (Figure 3B). These results indicated that elevated levels of IR and IGF1R enabled cancer cells to form IGF1R/IR HRs and increased their anti-proliferative response to figitumumab.

### N-linked glycosylation of IGF1R and IR in sensitive cells

**Figure 4:**
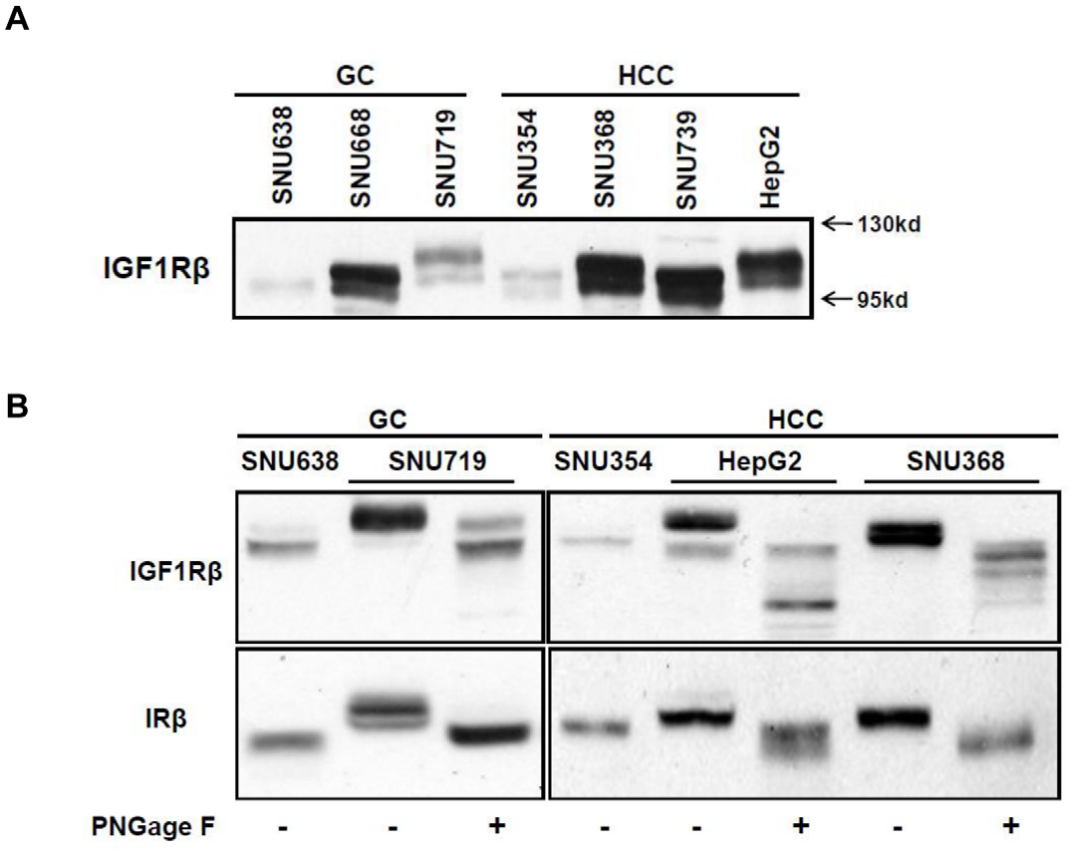
Analysis of NLG of IGF1Rβ and IRβ in figitumumab-sensitive cell lines. A) Immunoblot analysis of different IGF1Rβ migration pattern on SDS-PAGE. Electrophoretic mobility patterns of IGF1Rβ were analyzed in parallel by Western bloting. Experiments were repeated at least three times with similar results. B) Analysis of N-glycosylated IRβ and IGF1Rβ in sensitive cell lines by enzymic deglycosylation with PNGage F. All samples were incubated at 37°C for 12 h with PNGage F. IGF1Rβ and IRβ proteins were analyzed in parallel by Western blotting. The blots shown are representatives of three independent experiments.

Aside from the association between HRs and drug sensitivity, we also found that N-linked glycosylation (NLG) is an additional important factor that influences the response to figitumumab. Blotting for the anti-IGF1Rβ subunit revealed two isoforms around 95 and 105 kDa in most cells; however, sensitive cells showed a weak 105 kDa band and a stronger bands at 115 kDa (Figure 4A). In short, IGF1Rβ in figitumumab-sensitive cells migrated more slowly on SDS-PAGE than that in resistant cells. Interestingly, blotting for the anti-IRβ subunit produced the same band pattern as that of IGF1Rβ. In order to determine whether differences in molecular mass between sensitive and resistant cells were due to differences in N-glycosylation, we enzymatically deglycosylated IGF1R and IR with PNGage F, which removed all types of N-linked glycans. Treatment with PNGage F increased the electrophoretic mobility of both IGF1Rβ and IRβ in all figitumumab-sensitive cells (Figure 4B), indicating that IGF1Rβ and IRβ in the sensitive cells were mostly N-linked glycosylated.

### A specific NLG site of IGF1R in figitumumab-sensitive cells

**Figure 5:**
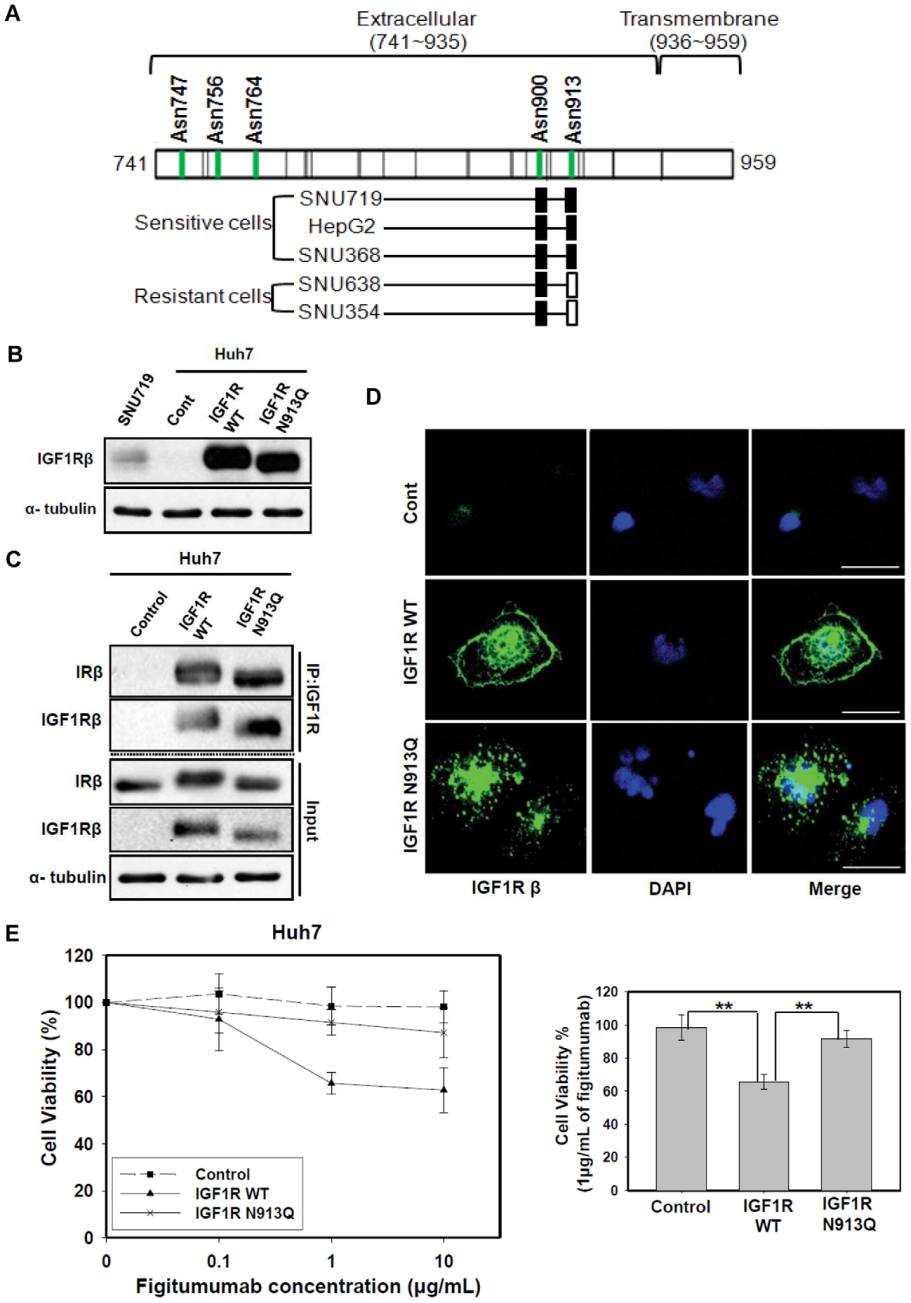
Identification of a specific N-linked glycosylation site (N913) of IGF1R in sensitive cell lines and its functional importance in the response to figitumumab. A) Identification of the NLG site occupancy of IGF1Rβ subunits. IGF1Rβ subunits containing N-linked glycosylation sites (Asn747, Asn756, Asn764, Asn900, and Asn913) were isolated from both drug sensitive (SNU719, HepG2 and SNU368) and resistance (SNU638 and SNU354) cells and identified by tandem MS by an increase of 1.0 Da from the corresponding mass of Asn as a result of conversion from N-linked glycosylated Asn to Asp. All the NLG at Asn900 in both sensitive and resistance cells were determined to be occupied with N-glycosylation (filled rectangle). NLG at Asn913 of the sensitive cell lines (HepG2, SNU719, and SNU368) were determined to be occupied with N-glycosylation (filled rectangle), whereas N-glycosites at Asn913 of the resistance cell lines (SNU638 and SNU354) were found to be unoccupied with N-glycosylation. (open rectangle). B) Effect of the N913Q site mutation on electrophoretic mobility patterns of IGF1Rβ. Huh7 cells (an IGF1R-negative cell line) were transfected with the empty pcDNA3.1(-) expression vector(Control), pcDNA3.1(-) containing wild-type IGF1R cDNA (IGF1R WT), or pcDNA3.1(-) with IGF1R mutation type cDNA (IGF1R N913Q). An equal amount of the cell lysate from the transfected cells was then subjected to Western blot analysis for IGF1Rβ. C) Effect of N913Q site mutation on the formation of IGF1R/IR heterodimeric receptors. An equal amount of the cell lysate from transfected cells was then subjected to immunoprecipitation (IP) with anti-IGF1R antibody followed by Western blot analysis for IRβ and IGF1Rβ. Input = total cell lysate without IP. D) Effect of N913Q site mutation on IGF1R localization. An immunofluoresence assay was conducted to observe the localization of IGF1R. IGF1R reactivity was visualized by confocal laser scanning microscopy (Scale bar: 30 µm). Representative images are shown. Green: IGF1R, Blue: nuclei. E) Effect of N913Q site mutation on figitumumab sensitivity. Huh7 cells transfected with empty pcDNA3.1(-) vector, vector containing wild-type IGF1R cDNA, or vector containing IGF1R (N913Q) mutation type cDNA were plated in 96-well plates and treated with increasing concentrations of figitumumab for 120 h (left). Cell viability percentages with 1 µg/mL figitumumab (right). Six replicate wells were included in each analysis, and at least three independent experiments were conducted. Data from replicate wells are presented as the mean of remaining cells. * *P-*values <0.05; ** *P-*values <0.01.

We next determined whether the variation of NLG of the IGF1Rβ subunit could be another candidate biomarker for figitumumab sensitivity. To identify a specific NLG site within the IGF1Rβ subunit, we used a combination of enzymatic de-glycosylation and mass spectrometry (MS) analysis. After evaluating the samples from both sensitive and resistant cells, we identified site-specific glycosylation at Asn900 and Asn913 among five putative NLG sites (Asn747, 756, 764, 900 and 913) of the IGF1Rβ subunit. Other NLG sites (Asn747, 756 and 764) were difficult to identify due to the presence of multiple NLG sites and the lack of proteolytic cleavage sites within the peptide sequence region (Figure 5A). Therefore, we focused on the Asn900 and Asn913 residues to evaluate site-specific NLG differences between the figitumumab-sensitive and resistant cells. A complete peptide fragmentation patterns of the tryptic peptide (^897^NPGNYTAR^904^) contained formerly N-glycosylated peptide at the Asn900 (an addition of +1 Da, N+1) was observed from both sensitive and resistance cells, which encompassed the Asn residue of the glycosylation site at Asn900 (Figure S7). These results demonstrated that Asn900 was glycosylated in both drug-sensitive and resistant cells. However, peptides with NLG at Asn913 were identified only in the sensitive, but not resistant cells, suggesting that this specific NLG site was not glycosylated in the resistant cells.

To further verify the NLG consensus site (N913) and its functional importance, a site-directed IGF1R mutant was constructed. Asn913 was replaced with a glutamine residue to yield an N913Q (Asn913 ln) mutant. To assess the functional consequences of this mutation, wild-type IGF1R and the mutant construct were transiently expressed in Huh7 cells. As shown in Figure 5B, the expression levels of wild-type and mutant IGF1R in the transfected cells were increased remarkably compared to cells transfected with the empty pcDNA3.1(-) vector. However, the N913Q mutation appeared as a 105 kDa band that migrated faster than the wild-type protein which produced a similar migration pattern of the protein on SDS-polyacrylamide gel electrophoresis (SDS-PAGE) in SNU719 cells. These observations confirmed that the ∼115 kDa band in sensitive cells corresponded to IGF1R that was NLG at N913.

Interestingly, it seems that removal of N-linked sugars from N913 of the IGF1R had no apparent effect on the formations of IGF1R/IR HRs. Rather, this mutation only affected on the NLG state of the receptor because HR levels were remarkably increased in the mutant IGF1R-transfected cells and the mutant receptor showed an increased migration rate on SDS-PAGE (Figure 5C). This result suggested that removal of the N-linked sugar from the N913 site altered the SDS-PAGE banding profile of IGF1Rβ but had no effect on the heterodimerization of IGF1R and IR.

### NLG regulates IGF1R localization to the plasma membrane and determines sensitivity to figitimumab

We next performed an immunofluorescence assay to determine whether mutation of the N913 consensus site prevented cell surface expression of IGF1R. Cells expressing wild-type IGF1R had an abundance of plasma membrane-bound IGF1R whereas the mutant form was primarily retained inside the cells with relatively little or no plasma membrane localization (Figure 5D). To assess the functionality of NLG-deficient-IGF1Rs compared to the wild-type form, we performed MTT assays. The results showed that the anti-proliferative effect of figitumumab was increased by overexpressing wild-type IGF1R, whereas cells transfected with the mutant IGF1R did not display any changes in drug sensitivity (Figure 5E). These results suggested that a lack of N-linked sugars at N913 in the IGF1R caused predominantly cytoplasmic localization of the receptor whereas wild-type IGF1R appeared to localize to the plasma membrane with increased sensitivity to figitumumab. Therefore, NLG at N913 appears to be essential for functional membrane-bound IGF1R and results in an increased response to anti-IGF1R antibody in cancer cells.

### Discussion

Figitumumab (CP-751,871) has been actively tested in patients with multiple myeloma, but the identification of biomarkers and mechanisms is needed to predict treatment responses and thus help with patient selection to maximize clinical benefits. Data from the present study suggest that the level of IGF1R/IR HRs can be a possible diagnostic biomarker for predicting sensitivity to anti-IGF1R antibody, particularly in GC and HCC cells. Previous studies have reported that the level of IGF1R itself may have predictive value in breast, lung, and colorectal cancers [31], [34]. In our study, however, neither expression of IGF1R alone nor levels of other IGF1R associated molecules, including IRS1, could be used to sufficiently predict figitumumab sensitivity (data not shown). Instead, we found that an important factor for the response to figitumumab seemed to be high expression levels of IR because only drug-sensitive cells showed high levels of IR as well as IGF1R (Figure 2A). Considering that several previous studies showed that overexpression of both IGF1R and IR may lead to an increased formation of IGF1R/IR HRs and expand the pool of IGF1 binding sites in various human malignancies [2], [3], [27], [35], we therefore focused on the concept that the level of IGF1R/IR HRs may be an important molecular biomarker for predicting figitumumab sensitivity. Consistent with these earlier reports, we observed that higher IR expression in the cells produced a greater number of endogenous HRs. Furthermore, figitumumab effectively disrupted IGF1-mediated IGF1R/IR HR formation, predominantly in cells overexpressing the HR. We also examined changes of IGF1R/IR HR levels in a mouse HepG2 xenograft model and determined that figitumumab reduced the expression of IGF1R/IR HRs (Figure S3B). This observation indicated that anti-IGF1R antibodies may preferentially act against cancer cells overexpressing IGF1R/IR HRs.

Although the physiological role of IGF1R/IR HRs is still unclear, a number of previous studies have indicated that they play major roles that may be more important than that of IGF1R [33]. This is because HRs, especially those containing IR-A hemidimers, have a broad binding specificity. IR-A expression up-regulates the IGF system by both increasing the affinity of heterodimers for IGFs and allowing insulin to activate the IGF1R in heterodimers [29]. In other words, overexpression of IR serves as a major mechanism of IGF1R signaling in cancer cells [36] by enabling the formation of more heterodimers which are available for binding ligands including IGF1, IGF2, and insulin. As a result, several studies have revealed the increased effectiveness of targeting heterodimeric receptors or simultaneously targeting both the IGF1R and IR as novel anti-cancer therapies compared to targeting IGF1Rs alone [30], [37], [38]. Moreover, our data extended previous studies indicating that monoclonal antibodies targeting both IGF1R and HRs markedly inhibit the growth of thyroid and breast cancer cells with high HR:IGF1R ratios [27]. Another study also showed that targeting IGF1R/IR HRs resulted in a more potent anti-tumoral response compared to antibodies targeting only IGF1Rs [30].

Aside from the association between HRs and figitumumab sensitivity, our results showed that NLG of the IGF1R and IR was another important indicator of drug sensitivity. We found that IGF1Rβ and IRβ showed an upward shifting on SDS-PAGE in all three figitumumab-sensitive cells compared to resistant cells (Figure 4A); the migration rate of these bands also increased following treatment with PNGage F (Figure 4B). These findings indicate that there was a variation in the addition of N-linked oligosaccharide to IGF1Rs in cancer cells. To verify this hypothesis, we identified a glycosylation site (N913) occupied by an N-linked sugar in only figitumumab-sensitive cells using a mass spectrometry approach. We also confirmed that NLG was required for efficient surface expression of IGF1R and sensitivity to figitumumab since removal of N-linked sugars via mutagenesis (N913Q) resulted in a predominantly cytoplasmic localization of the IGF1R and markedly reduced receptor translocation to the plasma membrane. These findings suggest that N913 in the IGF1R may be a specific glycosylation site needed for receptor translocation to the cell surface. Without post-translational NLG modification of the IGF1R at this site, IGF1R/IR HRs apparently fail to localize to the plasma membrane, thus preventing receptor-ligand binding and decreasing the efficacy of anti-IGF1R antibody-based cancer therapies.

Recently, a recent phase III trial of figitumumab administered in combination with carboplatin and paclitaxel failed to demonstrate survival benefit in advanced NSCLC patients. The study showed that the use of figitumumab with paclitaxel/carboplatin would be unlikely to improve overall survival compared to paclitaxel/carboplatin alone, mainly due to toxicity occurring in patients who randomly received figitumumab [15]. This study highlights the importance of selecting appropriate patients for clinical trials evaluating the anti-IGF1R antibody. Thus, additional studies identifying biomarkers for predicting the response to anti-IGF1R antibody are necessary. In this respect, our study may help identify a subset of cancer patients who would preferentially benefit from figitumumab therapy and provide important information for designing and conducting future clinical trials of figitumumab.

Although figitumumab failed to improve overall survival in the phase III trial, a subset analysis of this study offered clues about a potential predictive biomarker for predicting the response to figitumumab. In this analysis, patients with circulating levels of IGF1 of greater than 1 ng/mL experienced improved treatment outcomes including increased overall survival after receiving figitumumab with chemotherapy. More recently, the study by Gualberto et al. showed that higher pre-treatment levels of fIGF1 (>1 ng/mL) were predictive of the clinical benefit derived from the use of figitumumab with chemotherapy in NSCLC patients [39]. This group concluded that tumors which developed in a patient with high IGF bioactivity were more likely to become dependent on IGF1R signaling, and therefore may be more sensitive to figitumumab. This is not in contradiction with our data from experiments designed to identify potential predictive biomarkers of figitumumab sensitivity. Based on our finding, we suggest that heterodimerization of NLG IGF1R with IR in cancer cells may be a potential biomarker for predicting figitumumab sensitivity because cancer cells expressing more functional membrane-bound IGF1R/IR HRs that bind IGF1 ligands were more sensitive to treatment with anti-IGF1R antibody. Moreover, elevated levels of circulating IGF1 ligands might be associated with increased formation of IGF1R/IR HRs. Since increased levels of IGF1 ligand can mediate the formation of more IGF1R/IR HRs, preventing IGF bioactivity by blocking the interaction of IGFs with IGF1R/IR heterodimeric receptors, which play major roles in mediating the IGF/IGF1R signaling axis, might contribute to the anti-tumor activity of figitumumab in cancer cells dependent on IGF1R signaling. Therefore, we believe that functional membrane-bound IGF1R/IR HRs could be as important as fIGF1 levels for predicting sensitivity to anti-IGF1R antibody therapy.

In conclusion, data from the present study suggested that N-linked glycosylated IGF1R/IR HR levels can be a biomarker for predicting the response to figitumumab. To validate our results, similar experiments should be performed in preclinical and clinical settings. For example, we might be able to evaluate the levels of NLG IGF1Rs, IRs, and HRs in tissue samples from GC or HCC patients who respond favorably to figitumumab therapy using immunohistochemistry-based assays. This could confirm whether the level of functional membrane-bound IGF1R/IR HRs is an important predictor of sensitivity and responsiveness to targeted anti-IGF1R antibody-based therapy.

## Materials and Methods

### 

#### Cell Culture and Reagents

uman gastric cancer cells (SNU5, 16, 216, 484, 601, 620, 638, 668 and 719) and hepatocellular carcinoma cells (SNU354, 368, 423, 449, 739, and 886) were obtained from the Korea Cell Line Bank (Seoul, South Korea) [40], and HepG2 and Huh7 were purchased from the American Type Culture Collection (Manassas, VA). All cells were grown at 37°C with 5% CO_2_ in RPMI-1640 containing 10% fetal bovine serum (WelGENE Inc., Seoul. Korea). Figitumumab (CP-751,871) was provided by Pfizer Global R&D (CT, USA). A stock solutions (5 µg/mL) were stored at 4°C and diluted in fresh media before each experiment. Insulin growth factor 1 (IGF1) was purchased from Sigma-Aldrich (St. Louis, MO).

#### Growth Inhibition Assay

Tetrazolium dye (3-(4,5-dimethylthiazol-2yl)-2,5-diphenyltetrazolium bromide [MTT]; Sigma-Aldrich, St.Louis, MO) assays were used to evaluate the growth inhibitory effect of figitumumab. Cells were seeded in RPMI-1640 containing 10% fetal bovine serum in 96-well plates at a density of 3×10^3^ per well. After an over-night incubation, the cells were grown for 5 d in the presence of figitumumab (0, 0.1,1.0, and 10 µg/mL) at 37°C. After drug treatment, MTT solution was added to each well and the cells were incubated for 4 h at 37°C before the media were removed. Dimethyl sulfoxide (DMSO; 150 µL) was then added to each well, and the solution was shaken for 30 min at room temperature. Absorbance of each well was measured at 540 nm, using a microplate reader (Versa-Max, Molecular Devices, Sunnyvale, CA). Graphs were generated by nonlinear regression analysis of the data points to a four parameters logistic curve using SigmaPlot software (Statistical Package for the Social Sciences, Inc., Chicago, IL), and the IC30 value was calculated. Six replicate wells were included in each analysis, and at least three independent experiments were conducted.

#### Cell Cycle Analysis

Cells were plated in 60-mm dishes and grown to 50% confluence. After 24 h, the medium was exchanged with the test medium containing figitumumab (0, 0.1,1.0, and 10 µg/mL). After 48 h, the treated cells were harvested and fixed overnight with cold 70% ethanol at –20°C. After washing with PBS, the samples were incubated with 10 µg/mL RNase A (Sigma-Aldrich) and stained with 20 µg/mL propidium iodide (Sigma-Aldrich). Flow cytometric analysis (FACSCalibur flow cytometer; Becton Dickinson Biosciences, San Jose, CA) was performed; at least three independent experiments were conducted.

#### Protein Extraction and Immunoblotting

Cultured cells that had reached ∼70% to ∼80% confluence were used for protein analysis. The cells were washed with ice-cold phosphate-buffered saline (PBS) and lysed in radioimmunoprecipitation assay (RIPA) buffer (120 mM NaCl, 0.5% NP-40, 50 mM NaF, 0.2 mM PMSF, 0.1 mM pepstatin A, 0.2 mM leupeptin, 10 µg/mL aprotinin, and 1 mM benzamidine). Protein concentrations were quantified with a Bicinchoninic Acid Protein Assay Reagent (Pierce, Rockford, IL), according to manufacturer’s instructions. Samples containing equal amounts of total protein were resolved by sodium dodecyl sulfate-polyacrylamide gel electrophoresis (SDS-PAGE, 7%–12%) and transferred to nitrocellulose membranes (Whatman Protran, Dassel, Germany). The membranes were incubated in blocking solution containing 1% nonfat dry milk and 1% bovine serum albumin for 1 h at room temperature and probed overnight at 4°C with antibodies against p-IGF1Rβ (pY-1131/1146, dilution 1∶500), p-IRβ (pY-1361, dilution 1∶500), p-IRS(pS302, dilution 1∶500), p-STAT3 (pY-705, dilution 1∶1000), p-AKT (pS-473, dilution 1∶1000), p-ERK (pThr-202/Tyr-204, dilution 1∶1000), IGF1Rβ, IRβ, STAT3, AKT, and ERK, which were purchased from Cell Signaling Technology (Beverley, MA). Anti-IRS antibody was obtained from BD (San Jose, CA). Anti-α Tubulin was purchased from Sigma-Aldrich (St. Louis, MO). The membranes were then incubated for 1 h at room temperature with mouse and rabbit horseradish peroxidase-conjugated secondary antibodies (Pierce, Rockford, IL) diluted at 1∶3000 in T-TBS/1%BSA/1%dry skin milk.

#### Immunoprecipitation

Cells grown in 100 mm dishes were washed twice with ice-cold PBS and scraped into ice-cold lysis buffer (50 mM Tris-HCl [pH 7.4]), 120 mM NaCl, 0.5% NP-40, 50 mM NaF, 0.2 mM PMSF, 0.1 mM pepstatin A, 0.2 mM leupeptin, 10 µg/mL aprotinin, and 1 mM benzamidine). Lysates were centrifuged at 15000×g for 30 min at 4°C, The supernatants were removed and assayed for protein concentration. Lysis buffer (600 µL) containing equal amount of proteins were pre-cleared with protein A/G agarose beads (Upstate Biotechnology, Lake Placid, NY) and incubated overnight with anti-IGF1R antibody obtained from Santa Cruz Biotechnology (1∶100, Santa Cruz, CA) at 4°C with gentle rotation. Samples were then incubated with 50 µL protein A/G agarose beads (Upstate Biotechnology) for 2 h. The beads were washed four times with lysis buffer, collected by centrifugation, resuspended in 2×protein sample buffer, and boiled for 7 min at 100°C. Immunoprecipitated and total (input) protein samples were then resolved in SDS-polyacrylamide gel electrophoresis, and Western blotted with an anti-IRβ antibody and an anti-IGF1Rβ antibody.

#### siRNA for IGF1R Knockdown

Custom siRNA specific for IGF1R (target sequence: AACAATGAG TACAACTACCGC, sense strand: CAAUGAGUACAACUACCGCTT, antisense strand: GCGGUAGUUGUACUCAUUGTT), and negative control siRNA were obtained from Qiagen (Valencia, CA) and used to treat each cell line for 48h. The transfections were performed with LipofectAMINE ™ 2000 (Invitrogen, Carlsbad, CA) according to the manufacturer’s instructions. siRNA against IGF1R and negative control siRNA were used at a concentration of 60 nM.

#### ELISA

Star IGF1R and IR ELISA kits were purchased from Upstate Biotechnology. Proteins from all samples (50 µg/well) and quantification of IR and IGF1R were performed, according to the manufacturer’s protocol. All samples and standard were analyzed in duplicate. To quantify HRs, reagents from both the IR and IGF1R ELISA kits were used in combination. Anti-IR antibody-coated wells, IR protein standards, and the anti-IR detection antibody were used as standards for detecting HRs in the ELISAs. Absorbance was measured at 450 nm, using a microplate reader (Versa-Max, Molecular Devices).

#### Enzymatic Deglycosylation of the IGF1Rβ and IRβ Subunits

Enzymatic deglycosylation was performed with PNGase F [cat. no. R7884] purchased from Sigma-Aldrich (St. Louis, MO). Cultured cells were washed with ice-cold PBS and lysed in RIPA buffer. The lysates were then treated with 2 µL of 500 units/mL PNGase F and incubated at 37°C for 12 h. The reactions were stopped by heating to 100°C for 5 min. Samples containing equal amounts of total protein were then resolved on SDS-polyacrylamide denaturing gels (7%–12%) at a consistent voltage (80 V).

#### SDS-PAGE

Immunoprecipitated samples with figitumumab were loaded onto 4–12% SDS-PAGE gels, and run with MOPS/SDS running buffer (Invitrogen, Carlsbad, CA). The gel regions of interest were excised, in-gel digested, and extracted, as described previously [41]. Briefly, protein bands were excised and the cysteine residues were reduced with 15 mM TCEP (Sigma-Aldrich, St. Louis, MO, USA) and alkylated with iodoacetamide (Sigma-Aldrich). After dehydration with CAN, the proteins were digested with 30L of 12.0 ng/L modified porcine trypsin (Promega, Madison, Wi, USA) in 25 mM NH_4_HCO_3_, overnight at 37°C. Peptides were extracted with 60% v/v ACN in 1% formic acid, dried under vacuum. The dried peptide mixture was re-suspended in 50 mM NH_4_HCO_3_ and incubated with PNGase F (New England BioLabs, Ipswich, MA, USA) at 37°C for overnight. The deglycosylated peptide mixture was purified using a C18-desalting cartridge following the general protocol.

#### Mass Spectrometry Analysis

The dried peptide samples were dissolved in 20 µL 0.1% formic acid in H2O. The extracted peptide samples from the in-gel digestion were subjected to LC-MS/MS analysis on an LTQ-velos (ThermoFinnigan, San Jose, CA), coupled on-line with a nano-HPLC system (Proxyon, Copenhagen, Denmark), and equipped with a reversed-phase microcapillary electrospray ionization system [42]. 3 µL of the peptide mixtures were loaded onto the HPLC connected with an in-house, packed C18 column (10 cm length, 75 µm inner diameter). The peptides were sequentially eluted from the HPLC column with a gradient of 5 to 90% of buffer B (acetonitrile:water:formic acid, 98.5∶1∶0.5) in Buffer A (water:acetonitrile:formic acid, 98.5∶1∶0.5 [v/v/v/], at a flow rate of ∼0.2 µL/min. The eluted peptides were sprayed directly from the tip of the capillary column to the LTQ mass spectrometer for mass spectrometry analysis. The LTQ was operated in a data-dependent mode where the machine measured intensity of all peptide ions in the mass range of 400 to 1400 (mass-to-charge ratios). The top three most intense ions were isolated for collision-induced dissociation. Precursor ions were excluded after being targeted for MS/MS fragmentation after three scans in a 30 second period. Raw files were converted into mzXML files and peptides were assigned using SEQUEST [43] search against the human IPI database (version 3.80). All searches were performed with trypsin specificity allowing one missed cleavage. Cysteine modification with iodoacetamide was considered as fixed, oxidation of methionine, and 1 Dalton addition to asparagines as variable modification. The search considered a precursor ion mass tolerance of 1.5 Da, a fragment ion ass tolerance of 0.5 Da. Peptide assignments were validated using PeptideProphet [44], and the protein inference performed using ProteinProphet [45]. The list of protein identification s was filtered using a 0.9 probability threshold, which corresponds to less than 1% estimated false discovery rate.

#### Plasmid Constructs and Transient Transfection

Full-length IR cDNA (accession number BC117172) from pCR-XL TOPO (Thermo Scientific, Huntsville, AL) was isolated by double digestion with HindIII/XbaI (New England Biolab, Ipswich, MA) and subcloned into the mammalian expression vector pcDNA3.1- (Invitrogen). Full-length IGF1R cDNA (accession number BC113610) from pCR-XL TOPO was isolated by digesting with EcoRI (New England Biolab) and subcloned into pcDNA3.1-. To obtain mutant IGF1R cDNA, a point mutation converting an asparagine to glutamine (N913Q) was introduced into wild-type IGF1R cDNA using a QuickChange Site-Directed Mutagenesis kit (Stratagene. La Jolla, CA) according to the manufacturer’s protocol with the following primers: CACATCTCTCTCTGGGCAGGGGTCGACAGATC (forward) and GATCTGTCCACGACCCCTGCCCAGAGAGAGATGTG (reverse). IGF1R cDNA constructs were cloned into pcDNA3.1(-) plasmids resulting in wild type-IGF1R and mutant type-IGF1R (IGF1R WT and IGF1R N913Q). The mutant construct sequence was confirmed by site-directed sequencing using the following primers: TGAGGATCAGCGAGAATGTG (forward) and CAGAGGCATACAGCACTCCA (reverse). pcDNA3.1(-) vectors encoding the sequence for IR, wild type IGF1R, or mutant IGF1R(N913Q: AATCAG), were then used to transiently transfect into cancer cells. For transfections, 8 µL of LipofectAMINE ™ 2000 (Invitrogen) with 100 µL of serum- and antibiotic-free RPMI-1640 was added to 4 µg of pcDNA3.1(-) constructs harboring IR, wild type IGF1R, or mutant IGF1R (N913Q). After 20 min, the LipofectAMINE/cDNA solution was diluted with 4.8 ml of serum-free RPMI-1640 then incubated at 37°C in 5%CO_2_ for 6 h. The transfection medium was then replaced with complete culture media consisting of RPMI-1640, containing10% fetal bovine serum.

#### Immunofluorescence

In order to detect IGF1R localization, Huh7 cells grown on glass cover slips for 1 d were transfected with pcDNA3.1(-) vectors encoding the sequence for wild type IGF1R or mutant IGF1R (N913Q) for 48 h. After rinsing with PBS at room temperature, cells were fixed for 30 min with 3.7% paraformadehyde. After permeabilization with 0.1% Triton X-100 for 4–5 min, blocking was performed in 5% normal serum in PBS for 1 h at 37°C. The cells were then incubated overnight with anti-IGF1R antibody (1∶100, Cell Signaling Technology) at 4°C. After rinsing with PBS, the slides were incubated with conjugated goat anti-rabbit antibody (1∶1000, Invitrogen) for 1 h at room temperature. After washing with PBS, the cover slips were mounted onto glass slides in mounting reagent (DAKO, Glostrup, Denmark). All experiments were repeated three times. Digital images were acquired with a laser-scanning confocal microscope (Carl Zeiss, Jena, Germany) using appropriate lasers.

#### HepG2 Xenograft Model

All animal experiments were carried out in the animal facility of the Seoul National University in accordance with institutional guidelines. To determine the in vivo activity of figitumumab, 4-wk-old female BALB/c (nu+/nu+) athymic nude mice were purchased from Central Lab Animal Inc. (Seoul, South Korea) and were permitted to acclimatize to specific pathogen-free conditions for 1 wk before being injected with HepG2 cancer cells in 100 µL of PBS (1×10^7^ cells per 100 µL PBS). Figitumumab was diluted in PBS. The vehicle control group was given PBS alone. When the tumors reached a volume of 200 mm^3^, the mice were were randomly divided into groups (n = five mice per group) that received either vehicle (PBS) or figitumumab (125 µg/mL [6.3 mg/kg body weight] per mouse: once per wk) intraperitoneally. The tumor volume was determined by measuring the tumor mass every other day using calipers, and calculated according to the following formula: [(width)^2^×(height)^2^]/2. The general health of the mice and body weight were monitored at the time of tumor measurement. After the final treatment, all mice were euthanized according to institutional guidelines.

#### Statistical Analysis

A two-sided Student t-test was used as appropriate to compare tumor sizes in the xenograft-bearing mice. An unpaired two-tailed t-test was used to determine significant changes in cell viability and G1 arrest. Means ± SD are shown. All *P*-values <0.05 were considered statistically significant.

## Supporting Information

Figure S1 **Anti-IGF1R antibody (figitumumab) induced receptor internalization and degradation.** Time-dependent IGF1Rβ and IRβ protein degradation following figitumumab treatment. All cells (SNU719, SNU668, SNU638, SNU354, SNU368, SNU739, and HepG2) were treated with figitumumab (10 µg/mL) in complete medium at 37°C for the designated time periods. Cells were harvested at each time (1 h, 3 h, 6 h, 12 h, 24 h, 72 h) and lysed. The levels of IGF1R β and IR β proteins were analyzed in parallel by Western blotting. Representative blots from three independent experiments are shown.
(TIF)

Figure S2 **A) Effect of small-interfering RNA (siRNA) on IGF1R and IGF1R downstream molecules.** Custom iRNA specific for IGF1R (target sequence: AACAATGAG TACAACTACCGC, sense strand: CAAUGAGUACAACUACCGCTT, antisense strand: GCGGUA GUUGUACUCAUUGTT), and negative control siRNA were used at concentrations of 60 nM. SNU638, SNU719, SNU354, HepG2, and SNU368 cells were transfected with siRNA specific for IGF1R and negative control siRNA (60 nM). After 48 h, cell lysates were Western-blotted with the indicated antibodies. Representative blots from three independent experiments are shown. B) Effect of small-interfering RNAs (siRNA) against IGF1R on the anti-proliferative effect in sensitive cells. siRNA specific for IGF1R and negative control siRNA (60 nM) were used to transfect SNU638, SNU719, SNU354, SNU368, and HepG2 cells. After 48 h, cell were plated in 96-well plates and subjected to MTT assays. Mean values were derived from six replicates. Differences between the two groups were considered to be statistically significant (Bars = ±SE. *P-values <0.05; **P-values <0.01).
(TIF)

Figure S3 **Effect of figitumumab in** ***in vivo*** **mouse models.** A) Effect of figitumumab on activated IGF1R and IRS1 proteins in *in vivo* mouse models. After 1 d of figitumumab treatment initiation, the animals were sacrificed and the tumors were removed. The tumors were then homogenized by grinding the tumors in ice-cold lysis buffer to observe the changes in P-IGF1Rβ, IGF1Rβ, P-IRS1, IRS1, and α-tubulin protein expression. B) Effect of figitumumab on IGF1R/IR heterodimeric receptor levels in tumor tissues. On day 1 after figitumumab treatment, xenograft tumors were excised from euthanized mice from each group and snap frozen in liquid nitrogen. Tumors were then lysed with immunoprecipitation lysis buffer (50 mM Tris-HCl, pH 7.4) to detect changes in IGF1R/IR heterodimeric receptor levels. Samples were resolved in SDS-polyacrylamide denaturing gels (7.5%) with consistent voltage (80 V).
(TIF)

Figure S4 **Figitumumab recognizes IGF1R/IR heterodimeric receptors.** Lysates containing an equal amount of total protein (1 mg/mL) were immunoprecipitated with 1 µL of figitumumab (CP-751,871: 5 mg/mL) and Western-blotted with antibodies against IGF1Rβ and IRβ. Both IGF1Rβ and IRβ in SNU719, SNU368, and HepG2 cells were detected at high levels in the immunoprecipitates. The SNU601 cells, which showed modest sensitivity to figitumumab, also contained IGF1R/IR heterodimers. Representative blots from three independent experiments are shown.
(TIF)

Figure S5 **Anti-proliferative effect of figitumumab on MCF7 cells.** MCF7 breast cancer cells were used as a positive control for ELISA. The cells were treated with increasing concentrations of figitumumab (0, 0.1, 1.0, 10 µg/mL) for 120 hours to inhibit the growth of control cells by 30%. Six replicate wells were included in each analysis, and at least three independent experiments were conducted. The data from replicate wells are presented as the mean of the remaining cells. Bar = ±SE.
(TIF)

Figure S6 **Effect of figitumumab on insulin mediated IGF1R/IR heterodimeric receptors.** Figitumumab could not inhibit insulin-mediated signals or affect the formation of IGF1R/IR heterodimeric receptors. A) All cells were serum-starved for 24 hours, and then treated with insulin (100 nmol; 30 min) or figitumumab (10 µg/mL; 4 hours). SNU719 cells were incubated for 4 hour at 37°C with figitumumab followed by stimulation with insulin for 30 minutes. Total cellular extracts (1 mg) were extracted using IP buffer (pH 7.4), immunoprecipitated with anti-IR antibody, and Western blotted with anti-IGF1R antibody. The blot was then stripped and reprobed with anti-IRβ antibody to ensure equivalent loading of anti-IR antibody in all samples. B) Effect of figitumumab on insulin-mediated IGF1R signaling. SNU719 cells were serum-starved for 24 h and then treated with insulin (100 nmol; 30 min) or figitumumab (10 µg/mL: 4 h). The cell lysates were then Western-blotted with the indicated antibodies. Representative blots from three independent experiments are shown.
(TIF)

Figure S7 **MS/MS spectra of glycosylated peptides.** IGF1Rβ subunits containing N-linked glycosylation sites were isolated from both drug sensitive and resistance cells by immunoprecipitation using figitumumab. The IP samples were separated by SDS-PAGE and protein bands corresponding to the IGF1Rβ subunits were cut out and subjected to the in-gel digestion using trypsin. The resulting tryptic peptides were deglycosylated with PNGase F treatment. N-linked glycosylation sites were then determined by tandem mass spectrometry analysis by an increase of 1.0 Da from the corresponding mass of Asn as a result of conversion from N-linked glycosylated Asn to Asp. Major fragment ions referring to the a-, b-, and y- series are assigned, and the formerly glycosylated amino acid residues are underlined in the depicted peptide sequences. (A) MS/MS spectrum and sequencing results of an N-glycan-modified peptide corresponding to residues, ^896^LNPGNYTAR^904^ are shown. The expected increase in mass by N-glycan modification is 1.0 Da at Asn 900. The major fragment ions (a-, b-, and y-series) including N+1 (Asn900 plus 1.0 dalton) are consistent with N-glycosylation modification at Asn 900 (underlined). (B) MS/MS spectrum and sequencing results of an N-glycan-modified peptide corresponding to residues, ^905^IQATSLSGNGSWTDPVFFYVQAK^927^ are shown. The expected increase in mass by N-glycan modification is 1.0 Da at Asn913. The major fragment ions (a-, b-, and y-series) including N+1 (Asn913 plus 1.0 dalton) are consistent with N-glycosylation modification at Asn 913 (underlined).
(TIF)
