# Supplementary figures and images for: Heterodimerization of Glycosylated Insulin-Like Growth Factor-1 Receptors and Insulin Receptors in Cancer Cells Sensitive to Anti-IGF1R Antibody
Source: PLoS One. 2012 Mar 16;7(3):e33322. doi: 10.1371/journal.pone.0033322 (PMC3306383; doi:10.1371/journal.pone.0033322)

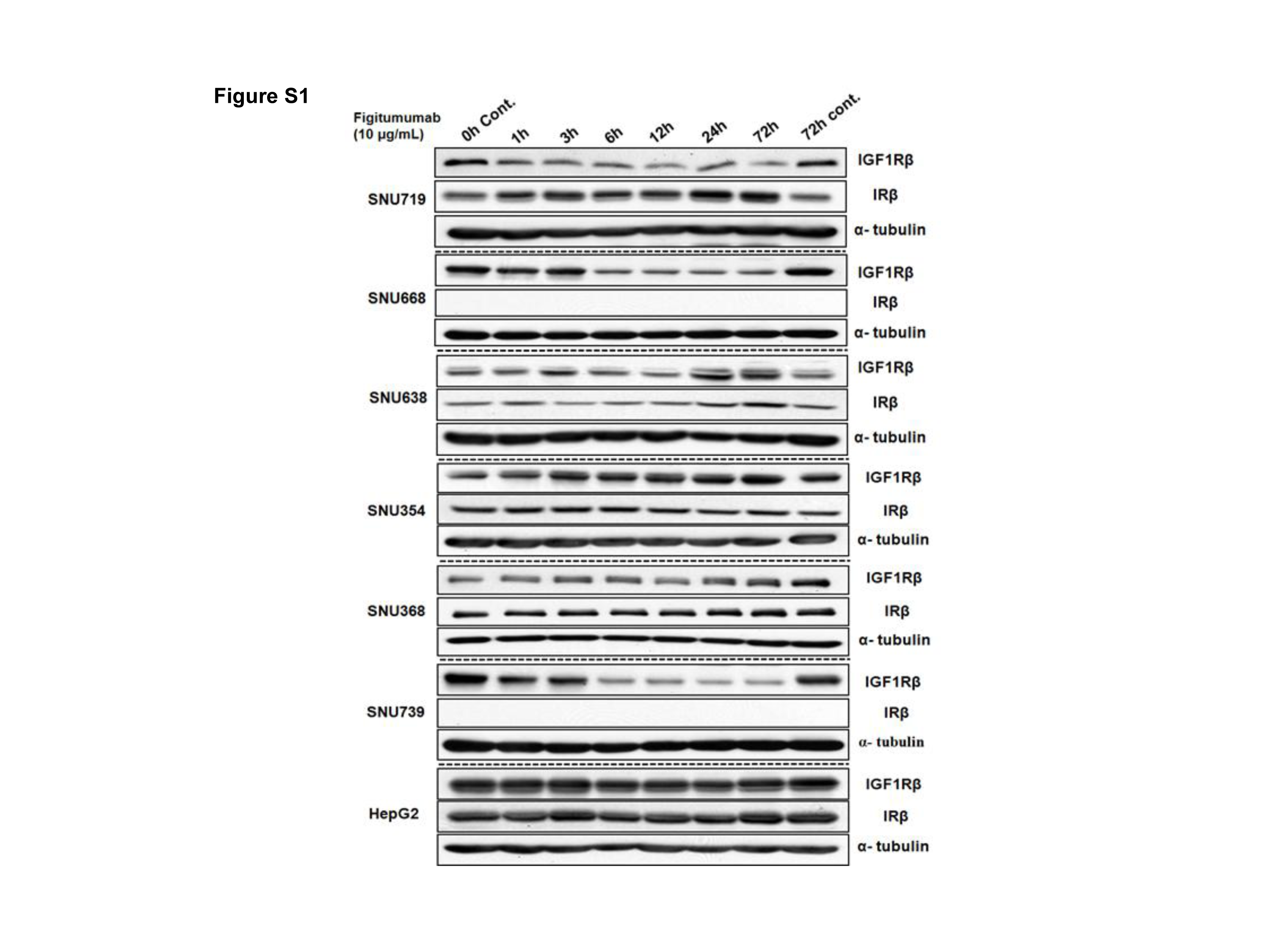

Supplement: Figure S1 — Anti-IGF1R antibody (figitumumab) induced receptor internalization and degradation. Time-dependent IGF1Rβ and IRβ protein degradation following figitumumab treatment. All cells (SNU719, SNU668, SNU638, SNU354, SNU368, SNU739, and HepG2) were treated with figitumumab (10 µg/mL) in complete medium at 37°C for the designated time periods. Cells were harvested at each time (1 h, 3 h, 6 h, 12 h, 24 h, 72 h) and lysed. The levels of IGF1R β and IR β proteins were analyzed in parallel by Western blotting. Representative blots from three independent experiments are shown. (TIF) [file pone.0033322.s001.tif]

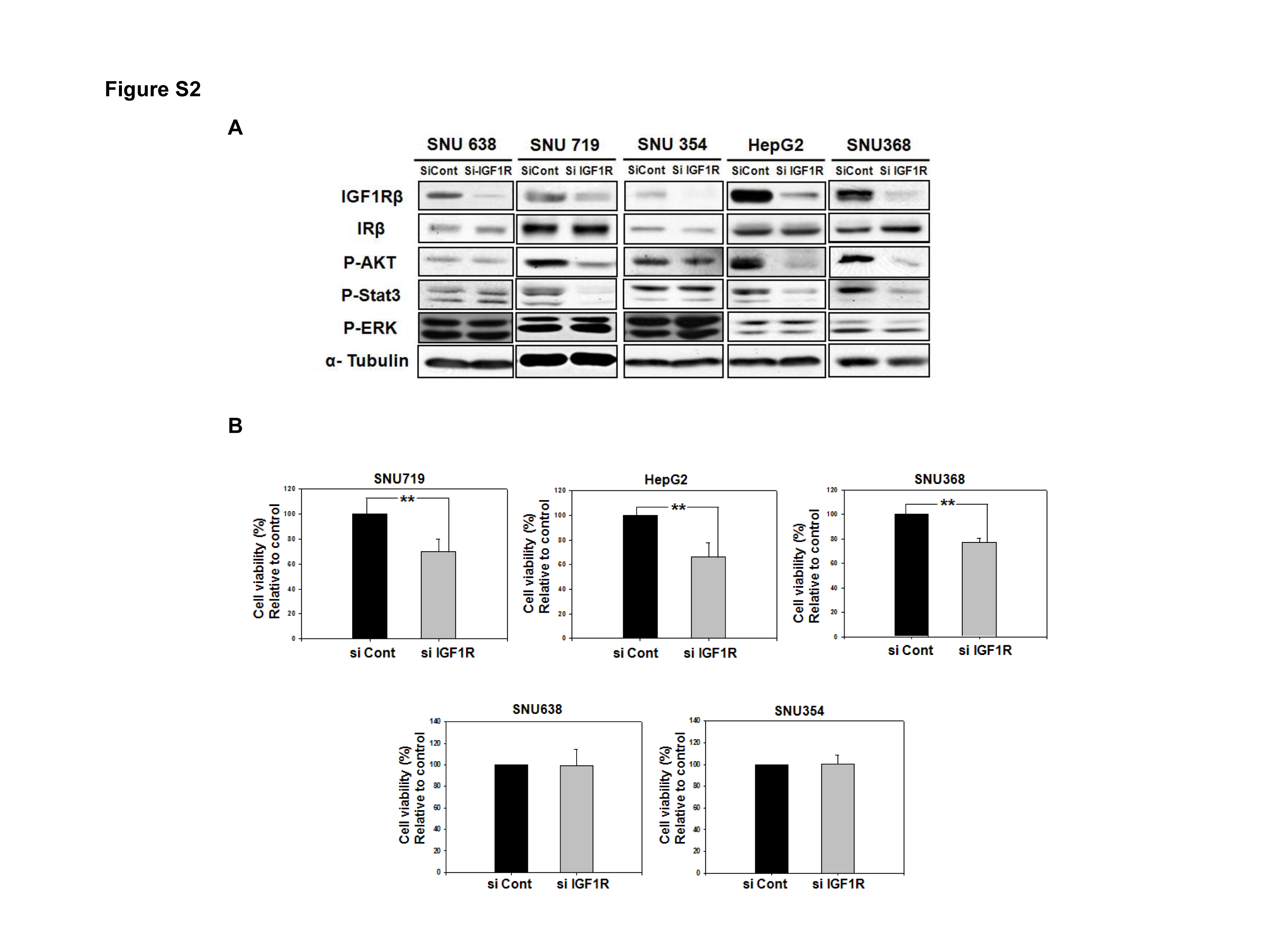

Supplement: Figure S2 — A) Effect of small-interfering RNA (siRNA) on IGF1R and IGF1R downstream molecules. Custom iRNA specific for IGF1R (target sequence: AACAATGAG TACAACTACCGC, sense strand: CAAUGAGUACAACUACCGCTT, antisense strand: GCGGUA GUUGUACUCAUUGTT), and negative control siRNA were used at concentrations of 60 nM. SNU638, SNU719, SNU354, HepG2, and SNU368 cells were transfected with siRNA specific for IGF1R and negative control siRNA (60 nM). After 48 h, cell lysates were Western-blotted with the indicated antibodies. Representative blots from three independent experiments are shown. B) Effect of small-interfering RNAs (siRNA) against IGF1R on the anti-proliferative effect in sensitive cells. siRNA specific for IGF1R and negative control siRNA (60 nM) were used to transfect SNU638, SNU719, SNU354, SNU368, and HepG2 cells. After 48 h, cell were plated in 96-well plates and subjected to MTT assays. Mean values were derived from six replicates. Differences between the two groups were considered to be statistically significant (Bars = ±SE. *P-values <0.05; **P-values <0.01). (TIF) [file pone.0033322.s002.tif]

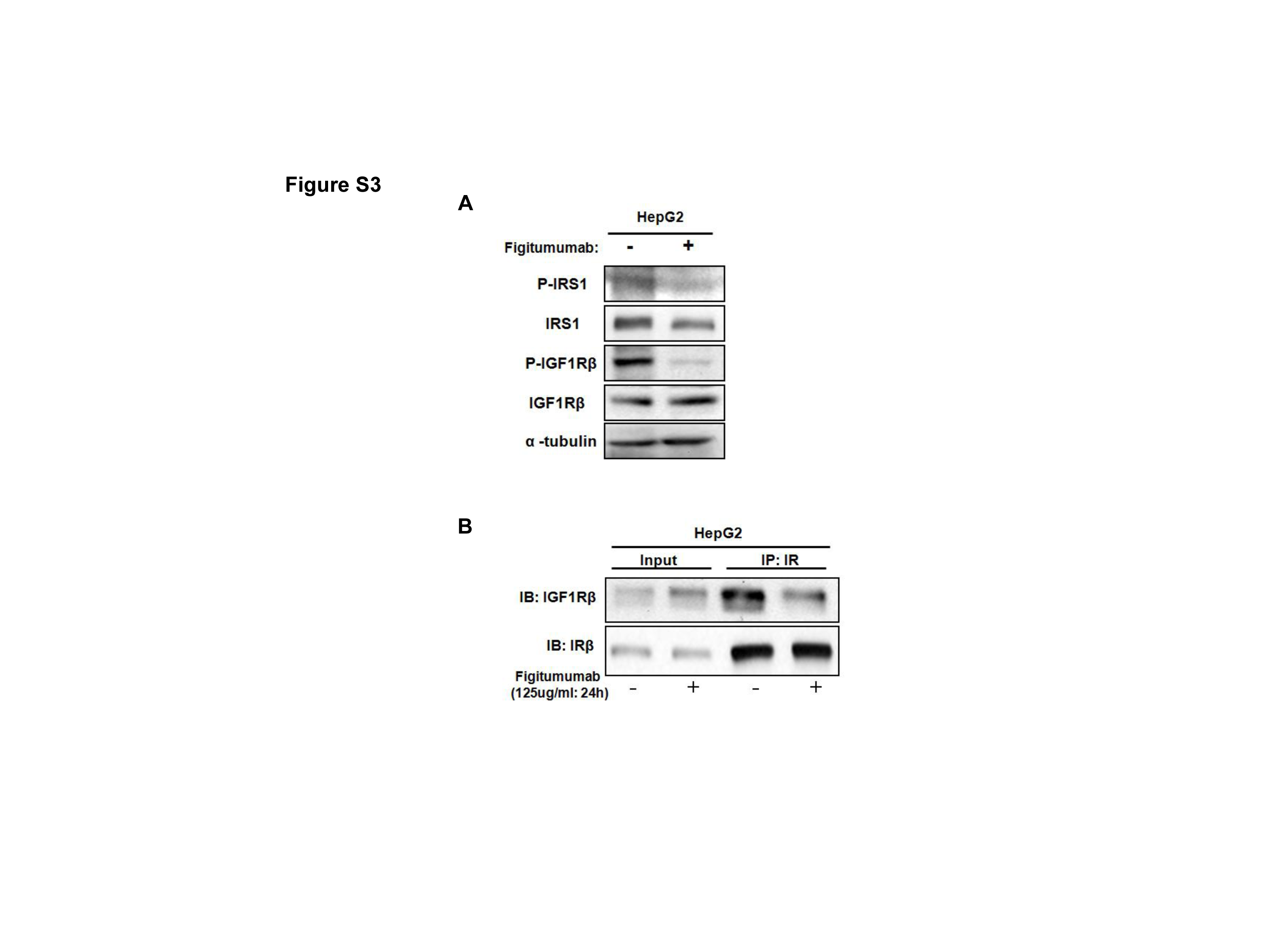

Supplement: Figure S3 — Effect of figitumumab in in vivo mouse models. A) Effect of figitumumab on activated IGF1R and IRS1 proteins in in vivo mouse models. After 1 d of figitumumab treatment initiation, the animals were sacrificed and the tumors were removed. The tumors were then homogenized by grinding the tumors in ice-cold lysis buffer to observe the changes in P-IGF1Rβ, IGF1Rβ, P-IRS1, IRS1, and α-tubulin protein expression. B) Effect of figitumumab on IGF1R/IR heterodimeric receptor levels in tumor tissues. On day 1 after figitumumab treatment, xenograft tumors were excised from euthanized mice from each group and snap frozen in liquid nitrogen. Tumors were then lysed with immunoprecipitation lysis buffer (50 mM Tris-HCl, pH 7.4) to detect changes in IGF1R/IR heterodimeric receptor levels. Samples were resolved in SDS-polyacrylamide denaturing gels (7.5%) with consistent voltage (80 V). (TIF) [file pone.0033322.s003.tif]

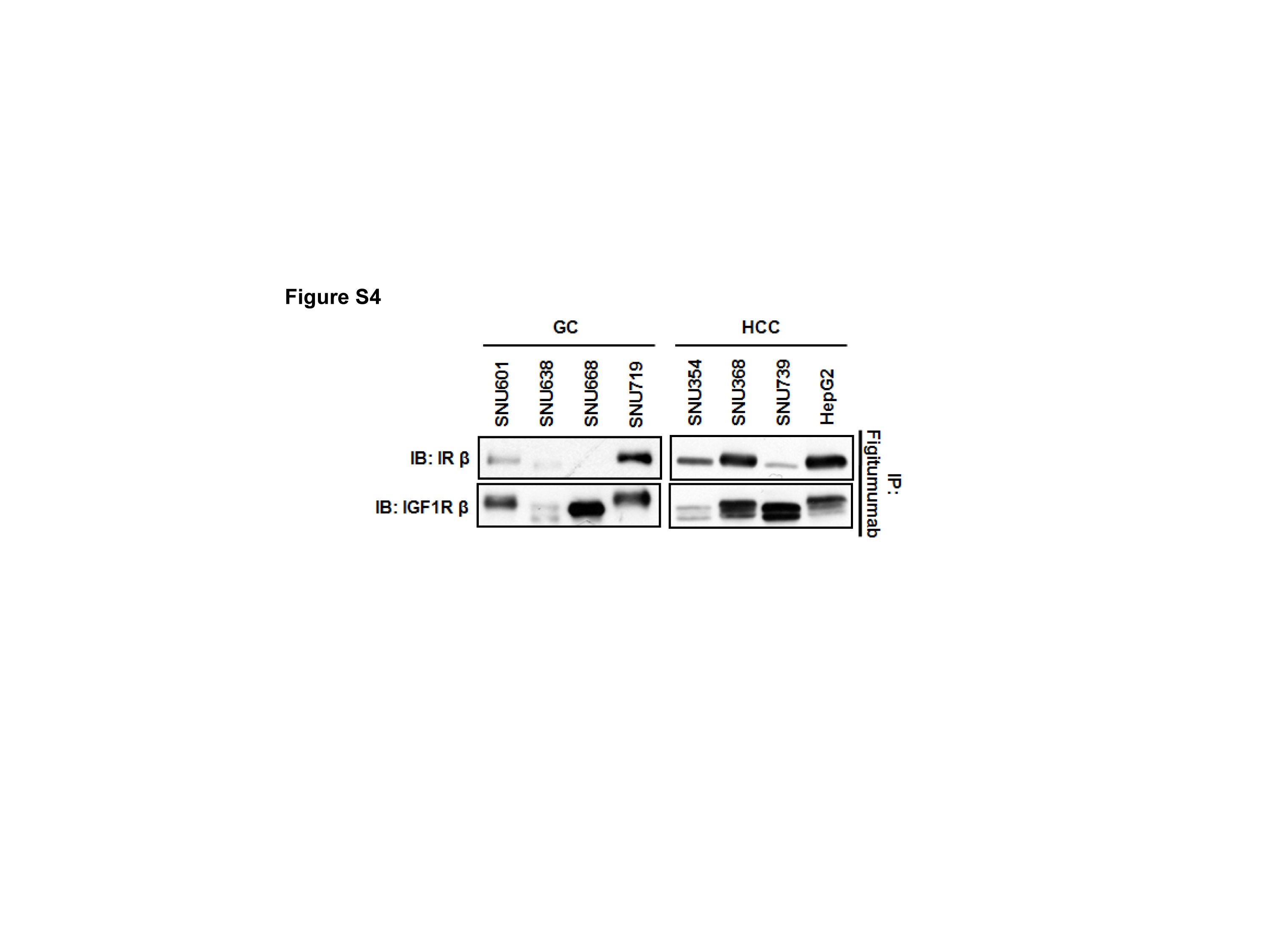

Supplement: Figure S4 — Figitumumab recognizes IGF1R/IR heterodimeric receptors. Lysates containing an equal amount of total protein (1 mg/mL) were immunoprecipitated with 1 µL of figitumumab (CP-751,871: 5 mg/mL) and Western-blotted with antibodies against IGF1Rβ and IRβ. Both IGF1Rβ and IRβ in SNU719, SNU368, and HepG2 cells were detected at high levels in the immunoprecipitates. The SNU601 cells, which showed modest sensitivity to figitumumab, also contained IGF1R/IR heterodimers. Representative blots from three independent experiments are shown. (TIF) [file pone.0033322.s004.tif]

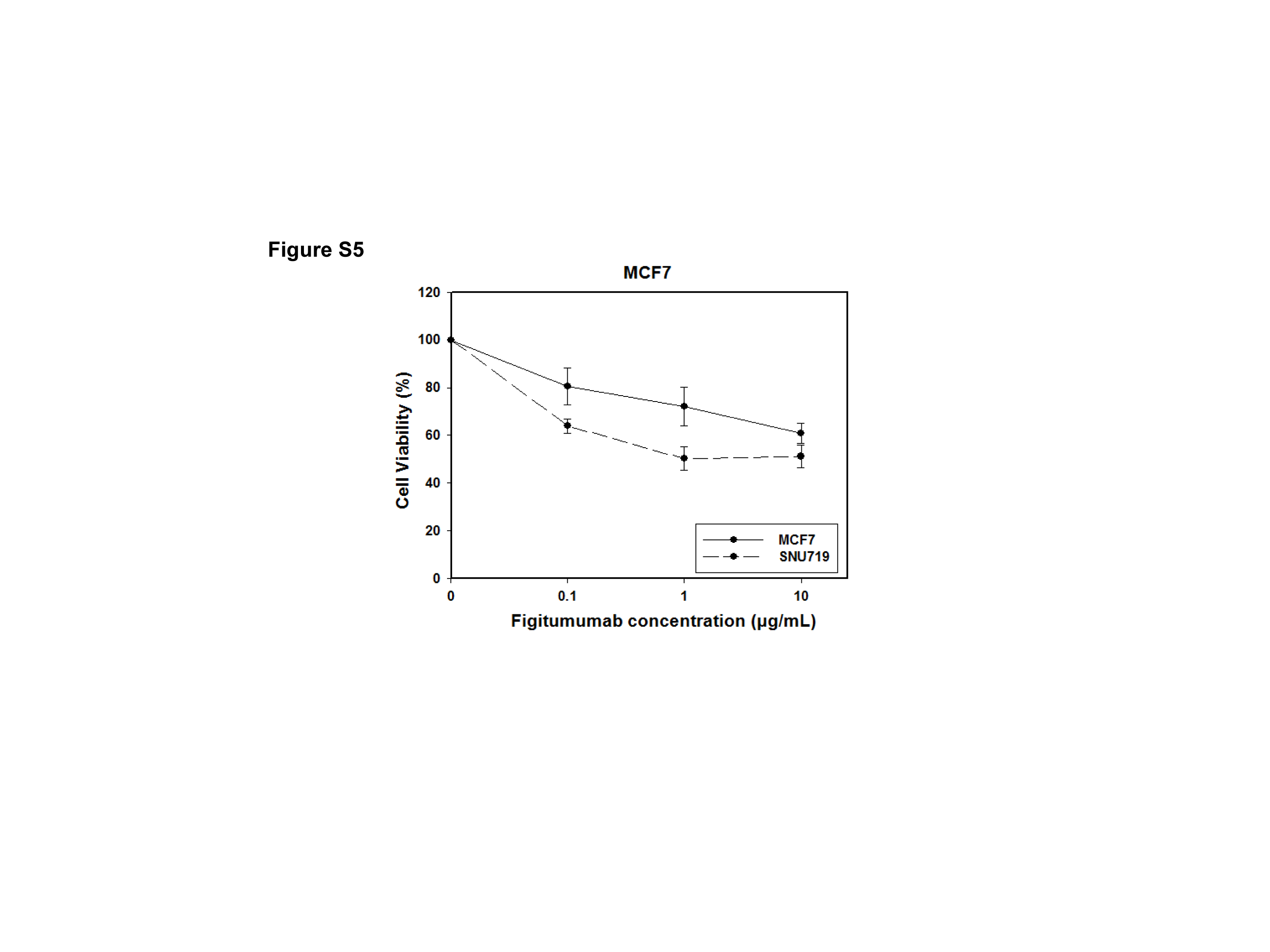

Supplement: Figure S5 — Anti-proliferative effect of figitumumab on MCF7 cells. MCF7 breast cancer cells were used as a positive control for ELISA. The cells were treated with increasing concentrations of figitumumab (0, 0.1, 1.0, 10 µg/mL) for 120 hours to inhibit the growth of control cells by 30%. Six replicate wells were included in each analysis, and at least three independent experiments were conducted. The data from replicate wells are presented as the mean of the remaining cells. Bar = ±SE. (TIF) [file pone.0033322.s005.tif]

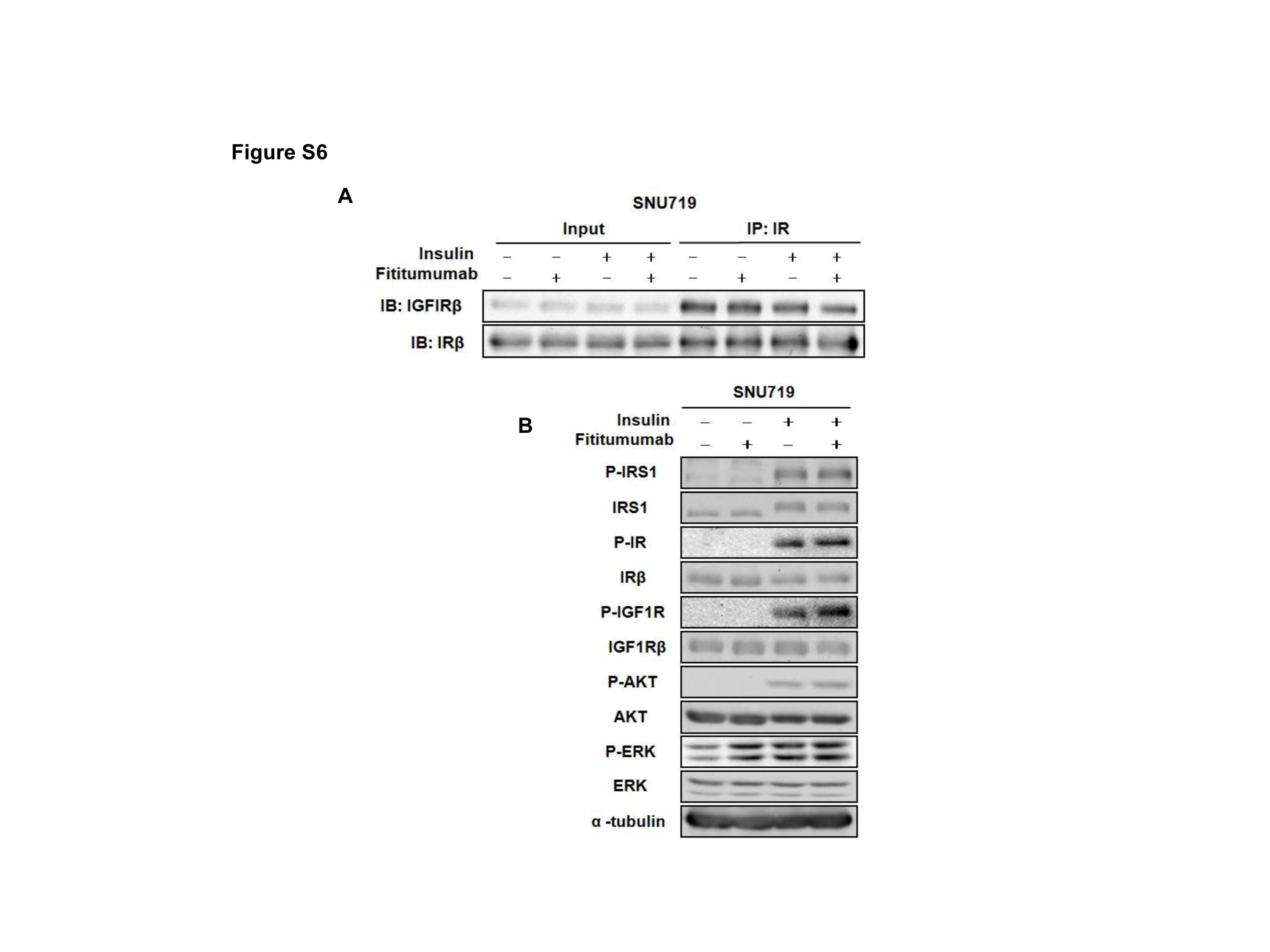

Supplement: Figure S6 — Effect of figitumumab on insulin mediated IGF1R/IR heterodimeric receptors. Figitumumab could not inhibit insulin-mediated signals or affect the formation of IGF1R/IR heterodimeric receptors. A) All cells were serum-starved for 24 hours, and then treated with insulin (100 nmol; 30 min) or figitumumab (10 µg/mL; 4 hours). SNU719 cells were incubated for 4 hour at 37°C with figitumumab followed by stimulation with insulin for 30 minutes. Total cellular extracts (1 mg) were extracted using IP buffer (pH 7.4), immunoprecipitated with anti-IR antibody, and Western blotted with anti-IGF1R antibody. The blot was then stripped and reprobed with anti-IRβ antibody to ensure equivalent loading of anti-IR antibody in all samples. B) Effect of figitumumab on insulin-mediated IGF1R signaling. SNU719 cells were serum-starved for 24 h and then treated with insulin (100 nmol; 30 min) or figitumumab (10 µg/mL: 4 h). The cell lysates were then Western-blotted with the indicated antibodies. Representative blots from three independent experiments are shown. (TIF) [file pone.0033322.s006.tif]

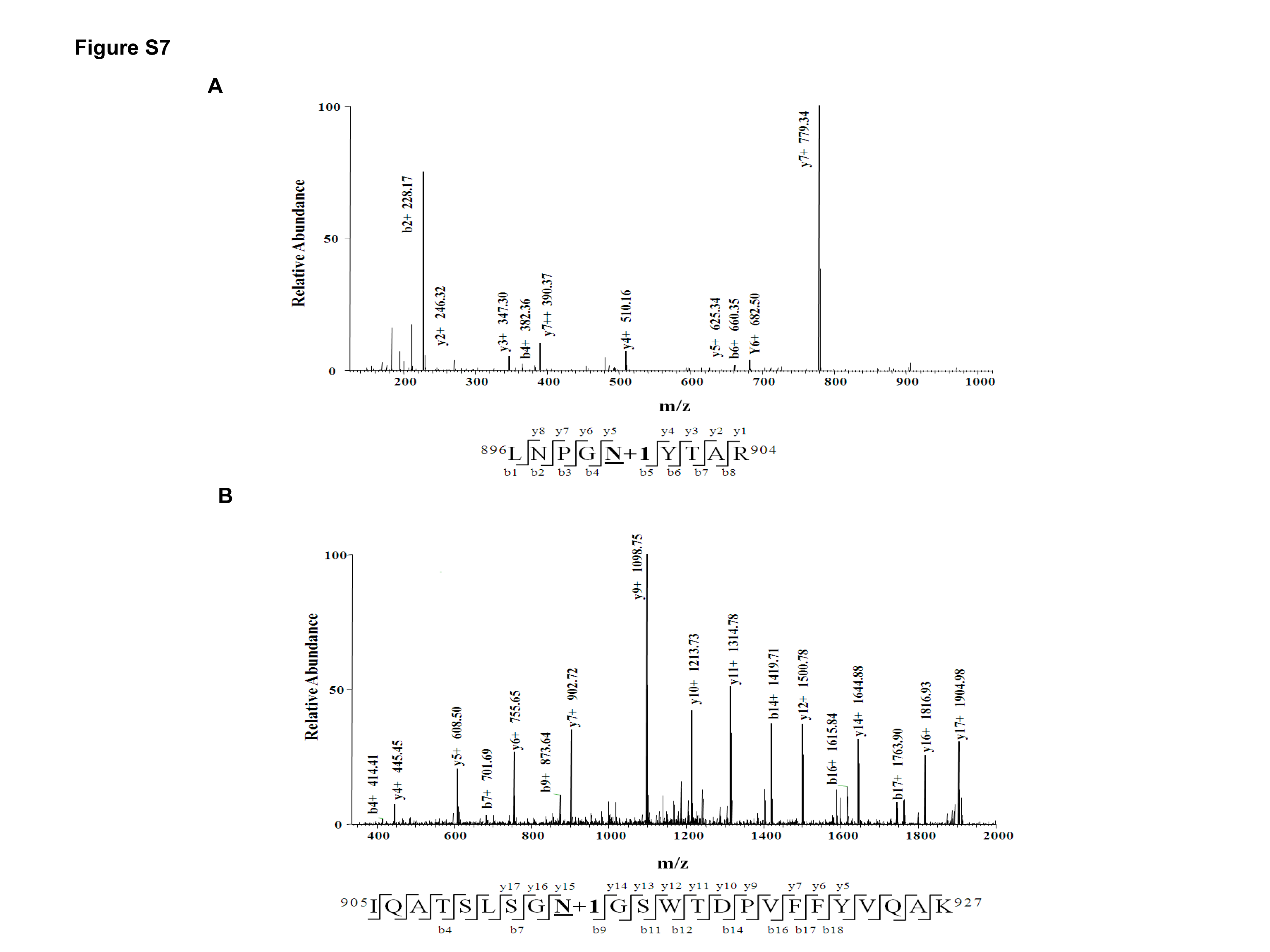

Supplement: Figure S7 — MS/MS spectra of glycosylated peptides. IGF1Rβ subunits containing N-linked glycosylation sites were isolated from both drug sensitive and resistance cells by immunoprecipitation using figitumumab. The IP samples were separated by SDS-PAGE and protein bands corresponding to the IGF1Rβ subunits were cut out and subjected to the in-gel digestion using trypsin. The resulting tryptic peptides were deglycosylated with PNGase F treatment. N-linked glycosylation sites were then determined by tandem mass spectrometry analysis by an increase of 1.0 Da from the corresponding mass of Asn as a result of conversion from N-linked glycosylated Asn to Asp. Major fragment ions referring to the a-, b-, and y- series are assigned, and the formerly glycosylated amino acid residues are underlined in the depicted peptide sequences. (A) MS/MS spectrum and sequencing results of an N-glycan-modified peptide corresponding to residues, 896LNPGNYTAR904 are shown. The expected increase in mass by N-glycan modification is 1.0 Da at Asn 900. The major fragment ions (a-, b-, and y-series) including N+1 (Asn900 plus 1.0 dalton) are consistent with N-glycosylation modification at Asn 900 (underlined). (B) MS/MS spectrum and sequencing results of an N-glycan-modified peptide corresponding to residues, 905IQATSLSGNGSWTDPVFFYVQAK927 are shown. The expected increase in mass by N-glycan modification is 1.0 Da at Asn913. The major fragment ions (a-, b-, and y-series) including N+1 (Asn913 plus 1.0 dalton) are consistent with N-glycosylation modification at Asn 913 (underlined). (TIF) [file pone.0033322.s007.tif]
